# Sex Differences in Synaptic Plasticity: Hormones and Beyond

**DOI:** 10.3389/fnmol.2018.00266

**Published:** 2018-07-31

**Authors:** Molly M. Hyer, Linda L. Phillips, Gretchen N. Neigh

**Affiliations:** Department of Anatomy and Neurobiology, Virginia Commonwealth University, Richmond, VA, United States

**Keywords:** sex differences, synaptic plasticity, hormones, mood disorders, stress response

## Abstract

Notable sex-differences exist between neural structures that regulate sexually dimorphic behaviors such as reproduction and parenting. While anatomical differences have been well-characterized, advancements in neuroimaging and pharmacology techniques have allowed researchers to identify differences between males and females down to the level of the synapse. Disparate mechanisms at the synaptic level contribute to sex-specific neuroplasticity that is reflected in sex-dependent behaviors. Many of these synaptic differences are driven by the endocrine system and its impact on molecular signaling and physiology. While sex-dependent modifications exist at baseline, further differences emerge in response to stimuli such as stressors. While some of these mechanisms are unifying between sexes, they often have directly opposing consequences in males and females. This variability is tied to gonadal steroids and their interactions with intra- and extra-cellular signaling mechanisms. This review article focuses on the various mechanisms by which sex can alter synaptic plasticity, both directly and indirectly, through steroid hormones such as estrogen and testosterone. That sex can drive neuroplasticity throughout the brain, highlights the importance of understanding sex-dependent neural mechanisms of the changing brain to enhance interpretation of results regarding males and females. As mood and stress responsivity are characterized by significant sex-differences, understanding the molecular mechanisms that may be altering structure and function can improve our understanding of these behavioral and mental characteristics.

## Introduction

Sex differences in behavior can be observed through both scientific inquiry and casual observations in social settings. Although gender roles have been proposed to account for some of the variability in behavior observed between males and females (Ruble et al., 1993; Aggen et al., 2011), behavioral differences extend beyond socially defined roles to physiological differences and certain well-established sexually dimorphic neural structures. Sex differences in neural structures have been documented in humans (Swaab and Fliers, 1985; Allen et al., 1989; Ruigrok et al., 2014; Catenaccio et al., 2016), but also in animals that do not have the social constructs of humans that define genders, including rodents (Gorski et al., 1978; Campi et al., 2013) and avian species (Nottebohm and Arnold, 1976; Balthazart et al., 2009). Some of the most notable sex differences in neural structure are evident in the hypothalamus of the mammalian brain (Gorski et al., 1978) and the song region in the avian brain (Nottebohm and Arnold, 1976). As these brain regions drive sex-specific reproductive-related behaviors, these findings are somewhat unsurprising. However, sex differences in neural structures extend beyond brain regions directly involved in reproduction and include structures linked to stress responsivity (for review see McEwen, 2010; Bekhbat and Neigh, 2018) and mood (for review see Bangasser and Valentino, 2014; Gobinath et al., 2015). Over the last three decades, technology and experimental design have advanced the wealth of knowledge regarding sex differences in the brain. Improved imaging techniques along with the elegant use of cell culture and pharmaceutical treatments, have further elucidated sex differences in structure (Phan et al., 2012; Farrell et al., 2015), connectivity (Ingalhalikar et al., 2014), signaling (Skucas et al., 2013; Harte-Hargrove et al., 2015), responsivity (Garrett and Wellman, 2009), and plasticity (Gould et al., 1990; Parducz et al., 2006). Sex differences are evident in adult neurogenesis, the birth of new neurons in adulthood (Ormerod et al., 2004; Tanapat et al., 2005; Mak and Weiss, 2010; Hyer et al., 2017). However, while sex-dependent modifications in adult neurogenesis can have a significant impact on synaptic plasticity (Galea et al., 2006; Livneh and Mizrahi, 2012; Vivar et al., 2016), that discussion is beyond the scope of this mini-review (see: Kempermann et al., 2015). Although these potential mechanisms are important to the understanding of sex differences in neural function, this mini-review focuses specifically on mammalian sex-differences in synaptic plasticity that may contribute to sex differences in neural function.

## Sex Differences in Structural Plasticity

**Figure 1:**
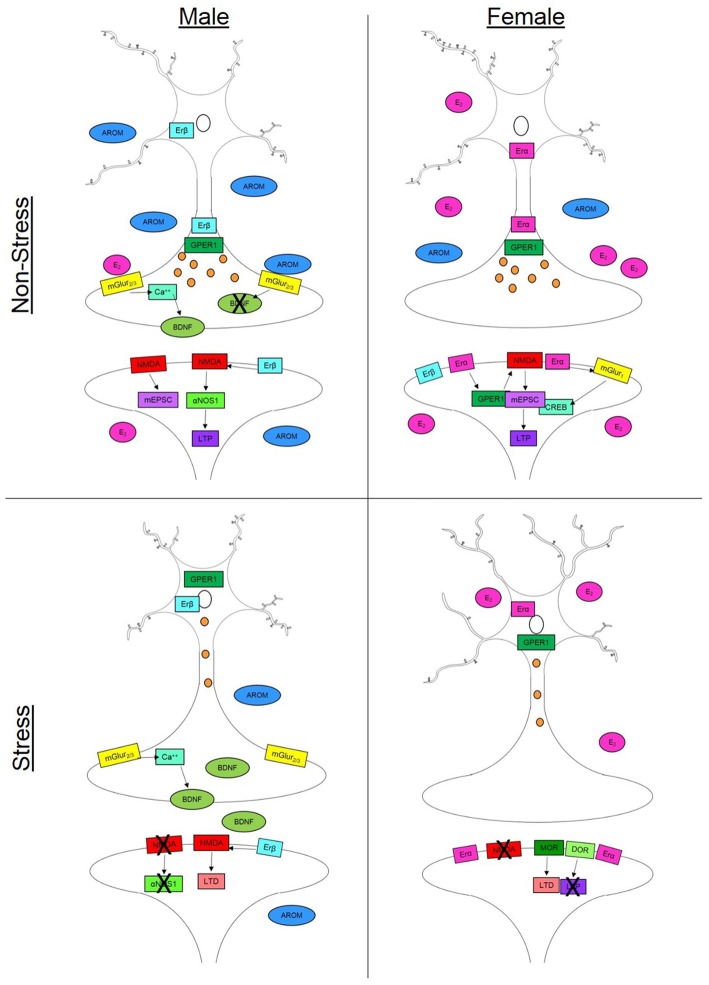
Synaptic plasticity is driven by a variety of sex-specific signaling mechanisms in males and females that can vary throughout the brain. In non-stress conditions, females (top right) have increased spine density compared to males in the hippocampus (top left) but decreased dendritic length in the prefrontal cortex (PFC). This increase in spine density in the female hippocampus can occur via multiple estradiol (E_2_)-dependent signaling mechanisms. Binding with estrogen receptor alpha (Erα) or the G-protein coupled estrogen receptor (GPER) can initiateN-methyl-D-aspartate (NMDA) channel signaling increasing mini excitatory postsynaptic currents (mEPCS) which ultimately drive long term potentiation (LTP). Estradiol can also act through Erα on metabotropic glutamate receptor 1 (mGluR1) which in turn drives cAMP response element-binding protein (CREB) phosphorylation in females. In males, NMDA activity is driven by activation of estrogen receptor beta (Erβ). This signaling cascade includes α nitric oxide synthase-1 (αNOS1) which drives LTP in males but not females. In the male presynaptic neuron, E_2_-dependent activation of mGluR_2/3_ initiates a calcium (Ca^++^) signaling cascade which facilitates the release of brain derived neurotrophic factor (BDNF). Testosterone (AROM) can alter the release of BDNF and other aspects of synaptic plasticity in the baso lateral amygdala. In stress conditions, males (bottom left) show decreased dendritic branching in the PFC but slight increases in spine density in the hippocampus. Possibly accounting for this increase is the reduction in circulating steroid hormones which can allow for increased BDNF release in males. Females (bottom right) on the other hand, experience increased dendritic length in the PFC and hippocampus as well as a suppression of spine growth in the hippocampus following stress. Females exhibit changes in opiate receptor (OR) signaling that can drive long term depression (LTD)—specifically through mu- and delta-OR activity. In the presynaptic neuron, axonal labeling of Erα and GPER1 facilitates vesicle transmission down the axon in both males and females. However, in stress conditions (bottom panels), both receptors migrate to the nucleus thereby reducing vesicle trafficking.

Spine density, sites for synaptic connections (Holtmaat and Svoboda, 2009), and dendritic arborization reflect changes in existing cell structure that can alter neural function and behavioral outcomes. Chronic stress results in differential patterns of dendritic remodeling in the hippocampus (Galea et al., 1997), prefrontal cortex (PFC; Garrett and Wellman, 2009; Farrell et al., 2015) and basolateral amygdala (BLA, Vyas et al., 2002). Clinical neuropsychiatric disorders related to stress exposure, for instance depression (reviewed in Qiao et al., 2016) and anxiety (reviewed in Leuner and Shors, 2013), occur concomitantly with changes in dendritic structure. The prevalence and incidence of depression and anxiety differ between the sexes (Piccinelli and Wilkinson, 2000; Naninck et al., 2011) and may be, in part, driven by sex-dependent dendritic and synaptic plasticity. Sex-differences have been noted in multiple dimensions of neuronal plasticity including neuron structure, dendritic arborization and spine density (Garrett and Wellman, 2009; Farrell et al., 2015) and sex steroids are sufficient to alter many of these parameters (Figure 1).

### Females

The influence of sex steroids on structural plasticity appears to be more pronounced in females, likely due to cyclical fluctuations in sex steroids. Concentrations of estrogens and progesterone fluctuate across the estrous cycle with peak levels appearing during proestrus and lower levels evident during estrus, metestrus and diestrus (Butcher et al., 1974). The human menstrual cycle shows fluctuations in ovarian steroids as well, with estrogen being lowest early on, then peaking at the end of the follicular phase to initiate ovulation. This peak is followed by a gradual surge in progesterone and a slight rise in estrogen throughout the luteal phase before both drop at the end of the cycle (Protopopescu et al., 2008; Catenaccio et al., 2016). Alterations in spine density are evident in response to changes in ovarian steroid levels across the estrous cycle in female rats. Spine density in area CA1 of the hippocampus peaks during proestrus when ovarian steroids are highest, then declines through the later stages (Woolley et al., 1990). Females generally have double the spine density of males, yet when they are ovariectomized no sex difference is evident, suggesting that the sex difference may be attributable to the activational effects of sex steroids (Gould et al., 1990; Shors et al., 2001). Ovariectomized females display a dramatic decline in spine density in the CA1 region of the hippocampus—a deficit which is reversed with estradiol or progesterone treatment in as low as 40 min (Gould et al., 1990; MacLusky et al., 2005). These changes in spine density appear to have functional implications, such that ovariectomized mice treated with low-dose estradiol demonstrate both improved learning and increased spine density in the CA1 region (Phan et al., 2012).

Sex differences in spine density are not limited to the hippocampus. For instance, female rats have more large spines in the nucleus accumbens than males (Forlano and Woolley, 2010). Sex differences in spine density have also been noted in the BLA, but somewhat surprisingly, testosterone, and not estradiol alone, appears to mediate these effects (Bender et al., 2017). The aromatase enzyme cytochrome P450 (AROM), present throughout the male and female BLA, is capable of converting testosterone to estradiol (Zhao et al., 2007). Administration of letrozole, an AROM inhibitor, to the BLA results in decreased spine density and eliminates long term potentiation (LTP) in females but not males (Bender et al., 2017). Females have greater excitatory synaptic input in the BLA compared to males, specifically in neurons located in the lateral and basal nuclei. The predominance of each nuclei shifts across the estrous cycle driving the balance of excitation-inhibition which is reflected through changes in BLA-dependent emotional memory across the estrous cycle (Blume et al., 2017). Collectively, these findings highlight the significant impact of sex steroids on the regulation of physiology and spine density in females; however, the following section discusses the impact of sex steroids on spine density that is also evident in males.

### Males

Although males do not produce estrogen or progesterone in as robust of concentrations as females, both sex steroids are present in males. The main source of estrogen in males is the conversion of testosterone to estrogen via aromatase. Gonadectomy in males reduces spine density in the CA1 region of the hippocampus, and this effect can be reversed through treatment with either testosterone or dihydrotestosterone (DHT). Based on the influence of estradiol demonstrated in females, one may hypothesize that estradiol would be equally efficacious in males; however, direct administration of estradiol is not sufficient to restore spine density in gonadectomized males (Leranth et al., 2003). Furthermore, testosterone appears to inhibit LTP and dendritic sprouting in the male hippocampus. Gonadectomy results in increased LTP and dendritic sprouting in CA3 mossy fibers which is dependent on increased brain derived neurotrophic factor (BDNF) signaling through the tyrosine kinase B (TrKB) receptor (Skucas et al., 2013). BDNF plays a well-known role in synaptic plasticity (Lu et al., 2014) and deficits in BDNF signaling in response to stress can be sex-dependent (Yamaura et al., 2013). Treatment with testosterone eliminated both the castration-induced increase in LTP and dendritic sprouting, suggesting that testosterone can act to inhibit BDNF-dependent structural plasticity (Skucas et al., 2013). Taken together, these data suggest that estradiol and testosterone regulate spine density in a sex- and region- dependent manner and that BDNF is a likely target mechanism for these hormones to modify synaptic connections.

### Sex Differences in Structural Response to Stress

Dendritic arborization and spine density are both modified by stress and are altered in individuals living with mood disorders. Males overall have more dendritic material than females (Juraska et al., 1985); however, females appear to have more dramatic shifts in dendritic architecture following mildly stressful experiences. For instance, females show increased dendritic length in the dentate gyrus (DG) following the mild stressor of exposure to a novel environment compared to males (Juraska et al., 1985). Interestingly, chronic stress will increase the length and complexity of dendritic arbors in females, possibly making them vulnerable to over-excitation (Farrell et al., 2015), but results in a reduction in dendritic arbors in males in the PFC. The increase is estradiol-dependent as ovariectomized female rats do not demonstrate the stress-induced increase in arborization (Garrett and Wellman, 2009). Chronic stress can also differentially drive subregion-specific plasticity. The dorsal hippocampus demonstrates enhanced markers of plasticity, specifically increased neuropeptide Y and ΔFOSB while the ventral region shows a decline in these same markers following chronic unpredictable stress (Hawley and Leasure, 2012). Further complicating our ability to understand sex differences in dendritic responses to stress, is the observation that the type of stressor impacts the outcome. Unlike chronic stress, acute foot shock generates the opposite effect on spine density in the hippocampus. Following acute foot shock, males have increased spine density in area CA1 of the hippocampus while females in diestrus demonstrate a reduction in dendritic spine density (Shors et al., 2001). Interestingly, regardless of the directionality, both males and females show no changes in spine density in response to acute stress if N-methyl-D-aspartate (NMDA) receptors are antagonized (Shors et al., 2004), suggesting a potentially unifying mechanism for the disparate consequence of stress. It is evident in female hippocampal slices that estradiol-induced LTP is dependent on an increase in the ratio of NMDA transmission to AMPA transmission (Smith and McMahon, 2005). Female rats that underwent inescapable shock to induce learned helplessness can be protected against the stress-induced loss of spines and LTP if treated with estradiol (Bredemann and McMahon, 2014). Thus, function of the hormone-dependent modifications of the NMDA receptor can dramatically alter the outcome of synaptic plasticity following stress.

## Sex Differences in Mechanisms of Synaptic Plasticity

### Estrogen Receptors

While changes in spine density and dendritic arborization are evident with cyclic fluctuations of hormones, the mechanisms that drive this structural plasticity are often at the molecular level. As estrogen has a significant impact on structural plasticity, much attention has been given to the mechanisms by which estradiol can modify neuron structure. Estrogens bind with estrogen receptor (Er) α, Erβ and G-protein coupled estrogen receptor 1 (GPER1). These receptors are localized throughout the brain of males and females providing estradiol a site of action to modify neuronal structure (Weiland et al., 1997). GPER1 is located throughout hippocampal neurons along the axon, dendritic tree, spine shafts, and is associated with vesicles in the terminal endings. Although anatomical distribution of GPER1 is similar between males and females, increased circulating estrogen concentrations in females leads to elevated axonal labeling of GPER1 in females as compared to males, suggesting alterations in vesicle transport with increased estradiol levels (Waters et al., 2015).

Unlike GPER1, ERα and ERβ exhibit more notable differences based on sex. Extranuclear ERα distribution in CA1 and CA3 at the ultrastructure level is primarily localized to the dendritic spine heads and at the base of spine shafts. Conversely, ERβ is localized to the soma and dendrite membranes (Mitterling et al., 2010). Regardless of circulating estrogen concentrations, females have higher densities of ERα than males (Shughrue et al., 1997; Mitterling et al., 2010), but ERα-immunoreactivity within females is sensitive to sex steroids concentrations. During diestrus, when circulating estrogen is at a comparatively low concentration, females demonstrate more ERα-immunoreactivity (ir) and ERβ-ir than that observed in proestrus females (Milner et al., 2001; Mitterling et al., 2010). Overall, elevated circulating concentrations of estradiol pair with reduced extranuclear-ir profiles of ERα and ERβ as compared to periods of low estrogen concentrations which are concomitant with elevations in extranuclear-ir (Mitterling et al., 2010). The shifting patterns of Er localization briefly summarized here, suggest a potential mechanism by which neuron structure could be differentially modified across the estrous cycle and lead to hormone-dependent plasticity in females.

While much of the benefits of estradiol are conferred to females, males also experience increased synaptic plasticity with estradiol signaling. Estradiol can drive potentiation of glutamatergic synapses in males as well as females (Teyler et al., 1980; Wong and Moss, 1992). However, the mechanisms by which the potentiation is initiated are sex-dependent. Estradiol elicits higher amplitude miniature postsynaptic excitatory currents (mEPSCs) in a synapse specific manner (Oberlander and Woolley, 2016). However, while similar results are evident in males, use of ER-selective agonists has identified a sex-dependent mechanism. In males, the potentiation is driven by ERβ while in females it is driven by GPER1. On the other hand, presynaptic potentiation is initiated by ERα in males but ERβ in females (Oberlander and Woolley, 2016).

Estradiol’s ability to alter function through Erα and Erβ (Walf and Frye, 2005; Boulware et al., 2013) is partially dependent on activation of metabotropic glutamate receptor 1a (mGluR1a; Boulware et al., 2013). The differing effects of Erα and Erβ are a result of receptor differences- specifically, differing N-terminal regions (Tremblay et al., 1997). This discrepancy results in activation of separate metabotropic glutamate receptors. Signaling through Erα activates mGluR1 which in turn drives cAMP response element-binding protein (CREB) phosphorylation. Erβ activation triggers mGluR2/3 signaling resulting in a downregulation of calcium mediated CREB phosphorylation (Boulware et al., 2005). It is also possible that Erβ activation may result in disinhibition of BDNF-releasing neurons (Blurton-Jones and Tuszynski, 2002) allowing for increased BDNF signaling to drive synaptic plasticity. As Erβ and BDNF are more prevalent in male synaptic plasticity, while Erα appears to drive female-specific plasticity, this may be a primary pathway for sex-dependent synaptic plasticity, allowing for rapid, non-genomic effects of estradiol on hippocampal plasticity (for review see Walf and Frye, 2006). Overall, these data show sex-specific mechanisms by which estradiol interacts with ERα and ERβ to mediate synaptic plasticity.

### NMDA Receptor Signaling

In addition to direct action through its own receptors, estradiol can modify NMDA receptor signaling to drive sex-specific plasticity. In female rats, estrogen-dependent increases in spine density are NMDA-dependent (Woolley and Mcewen, 1994). In gonadectomized females, estradiol benzoate treatment increases NMDA binding in area CA1 of the hippocampus compared to males. However, in males, baseline NMDAr binding is elevated in the DG compared to females (Romeo et al., 2005a). These findings suggest that estradiol’s interaction with NMDA receptors is dependent on the organizational effects of steroid signaling. DHT treatment to the hippocampus will increase NMDAr binding density in castrated male rats (Romeo et al., 2005b). Both testosterone and DHT have been shown to increase spinogenesis in males (Kovacs et al., 2003; Leranth et al., 2004) suggesting that activation of NMDA receptors by testosterone or its metabolites is necessary for spinogenesis in males while the same is true for estrogen in females. Furthermore, these sex-dependent mechanism may be exacerbated via cholinergic signaling as estradiol acts on NMDA receptors via cholinergic mechanisms in females but not in males (Volosin et al., 2006). Thus, NMDA receptor signaling is a necessary step for synaptic plasticity in both males and females, especially in response to stress (Shors et al., 2004), however the binding affinity and downstream effects are sexually dimorphic further complicating the interpretation of NMDA-dependent effects on synaptic plasticity.

### Nitric Oxide

Like its relationship with NMDA receptors, estrogen can act on synaptic plasticity indirectly through nitric oxide in a sex-dependent manner. LTP in males is dependent on α nitric oxide synthase-1 (αNOS1) signaling, however, in females LTP appears not affected in αNOS1 knockouts (Dachtler et al., 2012). This significant sex difference is likely due to the lack of NO and reduced NOS1 expression in the female hippocampus at baseline (Dachtler et al., 2012). However, estradiol application to female mouse derived tissue increases expression of NO in the female brain. Furthermore, NOS levels were altered across the estrous cycle, mirroring fluctuating estradiol levels—specifically, NOS levels were no different from males during proestrus and were lowest during diestrus (Hu et al., 2012). Thus, it is possible that NO-dependent LTP in females may be more likely during proestrous when estradiol is high and availability of NO is increased. However, it seems likely that NO-dependent LTP is a secondary mechanism by which LTP can be initiated in females, yet in males it is far more prominent.

### Opioid Receptors

Other mechanisms exist beyond steroid hormone-dependent plasticity, to drive changes in synaptic integrity. In proestrus, female rats have higher mossy fiber transmission in cultured hippocampal CA3 cells following administration of the generic opioid receptor antagonist naloxone. Cultured male tissue, on the other hand, does not show altered signaling. These data implicate the opioid receptor as an inhibitor in hippocampal transmission during peak estradiol levels in females. The mμ-opioid receptor antagonist Cys2, Tyr3, Orn5, Pen7-amide (CTOP) similarly enhances neural transmission, suggesting the mμ-opioid receptor is specifically down-regulating neural firing in females. Females also exhibit low frequency LTP during proestrus, suggesting that activity threshold is reduced when estradiol is elevated (Harte-Hargrove et al., 2015). Thus, a lowered activity threshold during times of peak estradiol supports enhanced learning and memory in proestrous females (Fernandez et al., 2008; Macbeth and Luine, 2010). However, concomitant inhibition of opioid receptors may be necessary to facilitate this estradiol-dependent benefit. Furthering this conclusion, electron microscopy has revealed more δ-opioid receptor-labeled spines in hippocampal pyramidal cells from proestrous females compared to males. Inhibition of δ-opioid receptors with the antagonist naltrinidole impairs low frequency LTP in proestrous females only. Taken together, these data implicate a sex-specific role of the δ-opioid receptor in LTP signaling in hippocampal pyramidal cells (Harte-Hargrove et al., 2015). As the opiate system can be activated by stress (Chaijale et al., 2013), understanding the role these signaling mechanisms play in plasticity can help define the pathways by which stress can modify synaptic plasticity in a sex-specific manner.

## Conclusion

It is evident that sex differences in synaptic plasticity are driven by both direct and indirect mechanisms of steroid hormones. While these mechanisms exist at the molecular and synaptic levels, they are further reflected in structural differences that ultimately result in sexually-dimorphic behaviors. Recent advances in human neuroimaging have provided insights into the translatability of these sex-differences in synaptic plasticity across mammalian species. Overall changes in white matter, gray matter and cerebral spinal fluid have been observed across the menstrual cycle in human women. Generally, gray and white matter volume appear to increase during the luteal phase when estrogen and progesterone are elevated in women (Catenaccio et al., 2016). Ruigrok et al. (2014) conducted a meta-analysis on gray matter volume and found significant sex-differences with men having more gray matter in the amygdala, hippocampus, parahippocampus, insula, and putamen (Catenaccio et al., 2016). Sex-differences in neural volume are further reflected in a region-specific pattern that fluctuates with the menstrual cycle—similar to what is observed across the rodent estrous cycle. The hippocampus is generally larger in men compared to women, however, during the luteal phase, when estrogen peaks, women see a significant increase in hippocampal volume (Protopopescu et al., 2008). Taken together, these data parallel findings from female rodents that sex-differences evident in certain neural structures are likely dependent on fluctuating hormones in women. Further research is needed to fully elucidate sex-differences in human neuroplasticity, however, it is clear that sex-differences are conserved across mammalian species. By understanding the synaptic mechanisms underlying behavioral differences between males and females, we can derive more information on the source of functional changes as well as their possible dysfunction when, and if, it occurs. Incorporating both males and females into these studies is essential given the extensive sex-differences in synaptic plasticity mechanisms and their functional outcomes.

## Author Contributions

MH outlined, researched and wrote this review article. In addition, she composed the figure. LP provided editorial comments and suggestions on the research areas to include. GN assisted in the drafting and editing of this manuscript and advised MH, a postdoctoral trainee, during the process.

## Conflict of Interest Statement

The authors declare that the research was conducted in the absence of any commercial or financial relationships that could be construed as a potential conflict of interest.
